# Spatiotemporal modeling of microbial metabolism

**DOI:** 10.1186/s12918-016-0259-2

**Published:** 2016-03-01

**Authors:** Jin Chen, Jose A. Gomez, Kai Höffner, Poonam Phalak, Paul I. Barton, Michael A. Henson

**Affiliations:** Department of Chemical Engineering, University of Massachusetts, 240 Thatcher Way, Life Science Laboratories Building, Amherst, MA 01003 USA; Department of Chemical Engineering, Process Systems Engineering Laboratory, Massachusetts Institute of Technology, Cambridge, MA 02139 USA

**Keywords:** Metabolic modeling, Bioreactors, Biofuels, Gas fermentation, Biofilms

## Abstract

**Background:**

Microbial systems in which the extracellular environment varies both spatially and temporally are very common in nature and in engineering applications. While the use of genome-scale metabolic reconstructions for steady-state flux balance analysis (FBA) and extensions for dynamic FBA are common, the development of spatiotemporal metabolic models has received little attention.

**Results:**

We present a general methodology for spatiotemporal metabolic modeling based on combining genome-scale reconstructions with fundamental transport equations that govern the relevant convective and/or diffusional processes in time and spatially varying environments. Our solution procedure involves spatial discretization of the partial differential equation model followed by numerical integration of the resulting system of ordinary differential equations with embedded linear programs using DFBAlab, a MATLAB code that performs reliable and efficient dynamic FBA simulations. We demonstrate our methodology by solving spatiotemporal metabolic models for two systems of considerable practical interest: (1) a bubble column reactor with the syngas fermenting bacterium *Clostridium ljungdahlii*; and (2) a chronic wound biofilm with the human pathogen *Pseudomonas aeruginosa*. Despite the complexity of the discretized models which consist of 900 ODEs/600 LPs and 250 ODEs/250 LPs, respectively, we show that the proposed computational framework allows efficient and robust model solution.

**Conclusions:**

Our study establishes a new paradigm for formulating and solving genome-scale metabolic models with both time and spatial variations and has wide applicability to natural and engineered microbial systems.

**Electronic supplementary material:**

The online version of this article (doi:10.1186/s12918-016-0259-2) contains supplementary material, which is available to authorized users.

## Background

Mathematical models of cellular metabolism are a complementary tool to experimentation for analyzing and engineering metabolic function. Over the past several decades, flux balance analysis (FBA) based on stoichiometric descriptions of cellular metabolism has emerged as the dominant approach for microbial metabolic modeling. FBA involves the formulation of stoichiometric equations describing the metabolic network followed by linear program solution of the underdetermined linear equation system subject to an assumed cellular objective such as growth rate maximization [1]. The advent of genome sequencing and bioinformatic technologies has allowed the reconstruction of large-scale metabolic networks in model organisms, which paved the way for the extension of FBA to genome-scale metabolic networks [2]. Curated genome-scale metabolic reconstructions are now available for a wide variety of microbial species, with new reconstructions announced on a weekly basis. Because genome-scale modeling is now an established tool, research has increasingly focused on novel ways to use these reconstructions for metabolic systems analysis and design.

Classical FBA methods assume time invariant and spatially homogeneous extracellular conditions and generate steady-state predictions consistent with well-mixed, continuous cultures [3]. Most microbial systems involve time and/or spatially dependent environments that should be incorporated within the metabolic description. The limitations of steady-state metabolic models have been addressed through dynamic extensions of stoichiometric models and classical FBA [4–7]. Dynamic flux balance models are obtained by combining stoichiometric equations for intracellular metabolism with dynamic mass balances on extracellular substrates and products under the assumption that intracellular metabolite concentrations equilibrate rapidly in response to extracellular perturbations [8]. The intracellular and extracellular descriptions are coupled through the cellular growth rate, secretion fluxes and substrate uptake kinetics, which can be formulated to include complex regulatory effects such as growth inhibition by metabolic byproducts. Dynamic flux balance modeling is now an established extension of FBA.

In contrast to the dynamic case, the development of metabolic models that account for spatially varying environments has received little attention. Such problems are very common in natural and engineered microbial systems. For example, naturally occurring microbial biofilms typically exhibit strong spatial gradients due to differential nutrient availability at the biofilm boundaries [9]. Spatial gradients are also present in synthesis gas bubble column reactors because dissolved CO and H_2_ concentrations decrease as the gas flows up the column due to cellular consumption [10]. The incorporation of genome-scale metabolic reconstructions within spatiotemporal models that account for both spatial and temporal variations in the environment is desirable to connect genes to metabolic phenotype and system function. For example, genome-scale metabolic reconstructions allow the effects of gene deletions and insertions in mutant strains to be directly investigated. Genome-scale spatiotemporal models have been solved using table lookups of precomputed FBA solutions [11–13], lattice based descriptions of nutrient diffusion [14, 15] and agent-based simulations [16]. These methods utilize a fixed time step over which the FBA linear program (LP) solution is assumed to remain unchanged and the ordinary differential equations (ODEs) representing the extracellular environment are integrated. By contrast, our approach allows the LP to be directly embedded within the ODEs and to be solved with variable time steps chosen by a stiff integrator. Therefore our computational framework represents an important step towards solving spatiotemporal models that combine a genome-scale description of intracellular metabolism and fundamental transport equations for the extracellular environment.

## Methods

### Model structure

The class of spatiotemporal metabolic models considered below is sufficiently general to encompass a wide variety of potential applications including microbial communities with interacting species and multiphase systems in which the liquid and gas phases move relative to each other. The framework is based on the standard dynamic flux balance modeling assumption that the intracellular metabolism is much faster than the extracellular dynamics, which we do not believe is any more restrictive when the environment exhibits spatial variations. Furthermore, we assume that spatial variations occur only in a single direction *z* for simplicity. Additional modeling assumptions include that constant gas and liquid phase volume fractions and velocities, constant gas–liquid mass transfer coefficients, constant cell and metabolite diffusion coefficients, and cell incompressibility. The last assumption allows a simple convection term to be used in the species mass balance equations. While cell compressibility could be included in the model if necessary, we expect that this effect would negligible under the low velocity liquid flows encountered in the examples consider here as well as in most practical applications.

Under these assumptions, a general set of model equations can be written as,

1 $$ \begin{array}{c}\hfill \frac{\partial {X}_i}{\partial t}=\left({\mu}_i-{\mu}_{di}\right){X}_i-\frac{u_L}{\varepsilon_L}\frac{\partial {X}_i}{\partial z}+{D}_{iX}\frac{\partial^2{X}_i}{\partial {z}^2}\hfill \\ {}\hfill \frac{\partial {M}_j}{\partial t}={\displaystyle \sum_{i=1}^N{v}_{ij}{X}_i-\frac{u_L}{\varepsilon_L}\frac{\partial {M}_j}{\partial z}+{D}_{jL}\frac{\partial^2{M}_j}{\partial {z}^2}+\frac{k_j}{\varepsilon_L}\left({M}_j^{*}-{M}_j\right)}\hfill \\ {}\hfill \frac{\partial {P}_j}{\partial t}=-\frac{u_G}{\varepsilon_G}\frac{\partial {P}_j}{\partial z}+{D}_{jG}\frac{\partial^2{P}_j}{\partial {z}^2}-\frac{k_j}{\varepsilon_G}\left({M}_j^{*}-{M}_j\right)\hfill \end{array} $$

The first equation represents a mass balances on the *i*-th microbial species where *X*_*i*_ is the biomass concentration, *μ*_*i*_ is the growth rate obtained from the genome-scale metabolic model, *μ*_*di*_ is the death rate, *u*_*L*_ is the liquid phase velocity, ${\varepsilon}_L$ is the liquid volume fraction and *D*_*iX*_ is the cellular diffusion coefficient that accounts for cell motility. The second equation represents a mass balance on the *j*-th liquid phase metabolite where *v*_*ij*_ is the net flux of metabolite *j* into the liquid phase from species *i*, *D*_*jL*_ is the liquid-phase metabolite diffusion coefficient, *k*_*j*_ is the gas–liquid mass transfer coefficient, and *M*_j_^*^ is saturation concentration in the liquid phase calculated from the associated gas-phase concentration using Henry’s law. The net flux *v*_*ij*_ is calculated as the difference between the synthesis rate obtained from the genome-scale metabolic model and the uptake rate calculated from Michaelis-Menten type kinetic expressions [17, 18]. The third equation represents a mass balance on the *j*-th gas phase component where *u*_*G*_ is the gas phase velocity, ${\varepsilon}_G$ is the gas volume fraction and *D*_*jG*_ is the gas-phase diffusion coefficient.

Boundary conditions for these equations are problem specific and can account for the supply/removal of liquid and/or gas phase components at the domain boundaries. Although not discussed here, the general model formulation can be extended to include a moving boundary as would be required for biofilm expansion. In the Appendix, two examples of formulated spatiotemporal metabolic models are presented: (1) a bubble column reactor for bacterial conversion of synthesis gas to ethanol; and (2) a bacterial biofilm associated with chronic wound infections. For the most part, these two models adhere to the general set of equations presented above. However, the species mass balance equation for the biofilm model (Equation 12 in Additional file 1: Appendix) has a slightly different formulation than the general equation to compensate for the lack of biofilm expansion in the model (see Additional file 1: Appendix).

### Model solution

Simulation of spatiotemporal metabolic models involves numerically solving a set of nonlinear partial differential equations (PDEs) with embedded linear programs. The efficient and stable solution of such models is a challenging problem at the forefront of microbial metabolic modeling. Our solution approach is based on spatial discretization such that the PDEs are converted into a large set of ordinary differential equations (ODEs) in time with embedded LPs. The spatial domain is discretized with *N* node points using an appropriate discretization method such as finite difference, finite volume or orthogonal collocation. If the original PDE model contains *N*_*X*_ microbial species, *N*_*M*_ liquid-phase metabolites and *N*_*P*_ gas-phase components, then the discretization procedure will yield a dynamic FBA model with *N*_*X*_ + *N*_*M*_ + *N*_*P*_ ODEs and *N*_*X*_ LPs at each node point.

Our approach for solving such large discretized models involves the use of DFBAlab [19], a MATLAB code that performs reliable and efficient dynamic FBA simulations. Widespread implementation of dynamic FBA has been hindered by numerical complications resulting from LPs becoming infeasible and having nonunique solution vectors. Infeasible LPs cause simulation failure as the right-hand side of the ODEs becomes undefined, and nonunique exchange fluxes cause this same right-hand side to become nonunique, producing an ODE system that integrators are unable to solve. These complications are addressed in our previous publication [20].

DFBAlab is a modified MATLAB implementation of our previously developed simulator [20]. DFBAlab reformulates the LP locally as an algebraic system, and it integrates a differential-algebraic equation system instead of ODEs with LPs embedded to increase speed. Hierarchical fixed-priority preemptive (lexicographic) optimization is used to determine uniquely all fluxes which appear in the right-hand side of the ODEs (i.e. exchange fluxes). All other fluxes not optimized lexicographically (i.e. internal fluxes) may still be nonunique, but their values do not affect the right-hand side of the ODEs. With lexicographic optimization, the right-hand side of the ODEs is guaranteed to be unique, allowing efficient and reliable integration. Finally, DFBAlab uses the Phase I LP of the simplex algorithm combined with lexicographic optimization to avoid infeasibilities.

More specifically, DFBAlab reformulates the FBA LP as a Phase I lexicographic LP to obtain all information required by the right-hand side of the ODEs as a unique vector-valued solution with the following order of objectives:

1. Minimize infeasibilities: If the first objective is equal to zero, the LP is feasible and all other objectives are consistent with the solution of the original FBA LP; otherwise, the objective is positive. If the original FBA LP is infeasible, the reformulated Phase I lexicographic LP still returns values for growth rate and exchange fluxes allowing the integration process to continue. This objective can be integrated to obtain a penalty function. This penalty function can provide useful insights on why and under what conditions the FBA model becomes infeasible.
2. Maximize growth rate: this is the traditional FBA objective.
3. Maximize/minimize all of the exchange fluxes appearing in the right-hand side of the ODEs. Each one of these objectives involves a linear combination of fluxes that can be minimized or maximized as appropriate. If there are *n* fluxes appearing in the right hand side of the ODEs, the vector-valued objective will require at most *n + 2* elements to obtain a unique right-hand side.

DFBAlab is designed to solve ODE systems; however, it provides a flexible framework that enables the solution of PDE models if the equations can be transformed into ODEs. Consider the following equation that describes the biomass concentration of the *i*-th species in a bubble column reactor:

2 $$ \frac{\partial {X}_i}{\partial t}=\left({\mu}_i-{\mu}_{di}\right){X}_i-{u}_L\frac{\partial {X}_i}{\partial z} $$

**Fig. 1:**
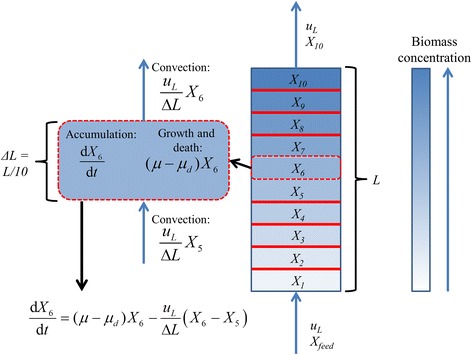
Discretization of the biomass concentration PDE for a single species in Equation 2. The bubble column reactor is divided into sections along the length dimension. Each section is represented by an ODE that has an accumulation term, a source/sink term due to bacterial growth and death, and two convection terms (in/out)

This PDE can be easily converted into an ODE by discretizing the spatial domain. If a simple backward difference formula is used to approximate the convection term, then the following set of ODEs is obtained for each point *j* in the spatial domain and each species *i*:

3 $$ \frac{\mathrm{d}{X}_{i,j}}{\mathrm{d}t}=\left({\mu}_i-{\mu}_{di}\right){X}_{i,j}-\frac{u_L}{\varDelta L}\left({X}_{i,j}-{X}_{i,j-1}\right), $$

where *L* is the length of the spatial domain, Δ*L* = *L/n* and *n* is the number of discretization points. In addition, *X*_*i,0*_ = *X*_feed_ = 0 and the outlet biomass concentration of the bubble column reactor is *X*_*i,n*_. A more detailed explanation for the single species case can be found in Fig. 1. A similar procedure is followed to obtain ODEs corresponding to the discretized PDEs of the liquid and gas phase components in (1). The flexibility of DFBAlab allows for the easy implementation and fast simulation of such discretized PDE systems. To ensure physically meaningful predictions for the two case studies presented below, the species growth rate μ_i_ and the secretion exchange fluxes *v*_*ij*_ were set equal to zero whenever DFBAlab detected that the LP for the i-th species was infeasible. This situation occurred when local nutrient uptake rates were insufficient to meet the non-growth ATP maintenance requirements. While this approach had the potential to make the right-hand side of the ODEs discontinuous, we found that DFBAlab had little problem integrating through such points because the growth rates and byproduct secretion rates tended to be very small immediately prior to an infeasibility occurring.

From a biological perspective, the additional objectives involving the exchange fluxes represent lower level cellular strategies than the main objective of growth rate maximization. The choice of these objectives is problem dependent and requires assumptions about the cellular metabolism. We typically assume that the cell regulation machinery is configured to maximize substrate uptake fluxes and minimize byproduct secretion fluxes, which is consistent with the main objective by maximizing the input of carbon containing and electron accepting metabolites and minimizing the output of carbon wasting byproducts. While DFBAlab requires specification of these lower level objectives, they impact the lexicographic optimization only when alternative optima occur. Our experiences with the two examples discussed in the following sections and other problems solved with DFBAlab is that the ordering of these objectives has a negligible impact on spatiotemporal model solutions because alternative optima typically occur only for short periods of simulation time. In other words, DFBAlab allows the integrator to reliably transition across short periods where alternative optima exist.

### Simulation codes

All simulations were performed with MATLAB 8.5 (R2015a) using DFAlab for dynamic flux balance model solution and Gurobi 6.0 for linear program solution. DFBAlab is freely available for both education and non-profit research purposes from https://yoric.mit.edu/dfbalab. Any entity desiring permission to incorporate this software or a work based on the software into commercial products or otherwise use it for commercial purposes should contact Dr. Paul Barton (pib@mit.edu). Simulation codes for the synthesis gas bubble column reactor and bacterial biofilm models can be obtained from www.ecs.umass.edu/che/henson_group/downloads.html.

## Results and discussion

### Spatiotemporal simulation of a synthesis Gas bubble column reactor model

An emerging route for the large-scale production of renewable fuels and chemicals is direct fermentation of waste gas streams and synthesis gas (syngas; mainly comprised of H_2_/CO/CO_2_) by specialized CO fermenting microbes. Because syngas can be produced relatively cheaply from a wide variety of biomass feedstocks [21, 22], the bottleneck in this route is the syngas fermentation step. Commercial development efforts are currently focused on bubble column reactors due to their superior gas–liquid mass transfer characteristics and enhanced operational flexibility [10]. Because CO and H_2_ concentrations decrease as the gas flows up the column due to cellular consumption, the column can have strong spatial gradients that affect cellular growth and product synthesis. The development of model-based techniques for simulating and optimizing these complex multiphase reactors is important to advance syngas fermentation technology.

#### 1. Bubble column model solution

The bubble column model was formulated by combining a genome-scale metabolic reconstruction of the syngas fermenting bacterium *Clostridium ljungdahlii* [23] with uptake kinetics for dissolved gases and reaction-convection–dispersion type equations for gaseous and dissolved substrates and synthesized metabolic byproducts. Our preliminary FBA calculations with the typical maximum growth objective showed that the only metabolic byproducts for growth on CO/H_2_ mixtures were ethanol, acetate and CO_2_. While other byproducts could be secreted under bubble column operating conditions, we did not attempt to determine or model other byproducts due to our focus on ethanol production. Therefore, the spatiotemporal metabolic model was comprised of 9 PDEs for the liquid-phase concentrations of *C. ljungdahlii* biomass, ethanol, acetate, CO, H_2_ and CO_2_ and the gas-phase concentrations of CO, H_2_ and CO_2_ (see Additional file 1: Appendix). Model parameters were obtained from the literature to the extent possible with the remaining parameters specified within reasonable ranges (Table 1). The interested reader is directed to our other paper [24] for additional details about the bubble column model formulation and model sensitivity to various column operating and substrate uptake parameters.

**Table 1:** Parameter values for the synthesis gas bubble column reactor model

Parameter	Value	Parameter	Value
*L*	25 m	*v*_*max,CO*_	35 mmol/gDW/h
*A*	5 m^2^	*K*_*m,CO*_	0.02 mmol/L
*u*_*G*_	75 m/h	*v*_*max,H2*_	70 mmol/gDW/h
*u*_*L*_	0.25 m/h	*K*_*m,H2*_	0.02 mmol/L
*D*_*A*_	0.25 m^2^/h	*v*_*max,CO2*_	35 mmol/gDW/h
*T*	37 °C	*K*_*m,CO2*_	0.02 mmol/L
*P*_*L*_	1.013x10^5^ Pa	*K*_*I*_	10 g/L
*x*_*C*_	0.6	*C*_*GF*_	80.64 mmol/L
*x*_*H*_	0.4	*H*_*GF*_	53.76 mmol/L
*x*_*D*_	0	*D*_*GF*_	0 mmol/L
*H*_*C*_	8×10^−4^ mol/L/atm	*X*_*0*_	0.1 g/L
*H*_*H*_	6.6×10^−4^ mol/L/atm	*C*_*G0*_	80.64 mmol/L
*H*_*D*_	2.5×10^−2^ mol/L/atm	*H*_*G0*_	53.76 mmol/L
*k*_*m,C*_	80 h^−1^	*D*_*G0*_	0 mmol/L
*k*_*m,H*_	200 h^−1^	*C*_*L0*_	1.642 mmol/L
*k*_*m,D*_	80 h^−1^	*H*_*L0*_	0.903 mmol/L
ε_*G*_	0.0646	*D*_*L0*_	0 mmol/L
ε_*L*_	0.9354	*E*_*L0*_	0 g/L
		*A*_*L0*_	0 g/L

The convection terms were discretized using an upwind finite difference approximation with third-order accuracy due to its well established numerical accuracy and stability properties for convection dominated problems [25]. We found that the addition of axial dispersion terms to the liquid phase mass balances greatly improved numerical stability of the model (see Additional file 1: Appendix), as has been well documented in other applications [25]. These dispersion terms were discretized using a central difference approximation with second-order accuracy. Because the upwind formula was not implementable at the reactor boundaries, a first-order backward difference approximation was used at these locations. The discretization procedure yielded a set of 9 ODEs at each node point.

The lexicographic optimization objectives required by DFBAlab were specified to reflect the known or expected physiology of *C. ljungdahlii* (Table 2). We found that the ordering of these objectives had no noticeable effect on predicted metabolic phenotypes and bubble column behavior. Each node point was represented by 9 ODEs for the biomass and biochemical species concentrations, 3 algebraic equations for the local dissolved gas uptake rates and 6 LPs for lexicographic optimization. We typically employed 100 node points to obtain a nearly converged solution using DFBAlab combined with the LP solver Gurobi and the stiff ODE solver ode15s.

**Table 2:** Cellular objective functions used for *C. ljungdahlii* metabolism

Number	Direction	Objective	Reason
1	Maximize	Growth rate	Assumed primary objective
2	Maximize	CO uptake rate	Maximize nutrient consumption
3	Maximize	H_2_ uptake rate	Maximize nutrient consumption
4	Minimize	CO_2_ synthesis rate	Minimize byproduct synthesis
5	Minimize	Acetate synthesis rate	Minimize byproduct synthesis
6	Minimize	Ethanol synthesis rate	Minimize byproduct synthesis

#### 2. Prediction of bubble column performance

**Fig. 2:**
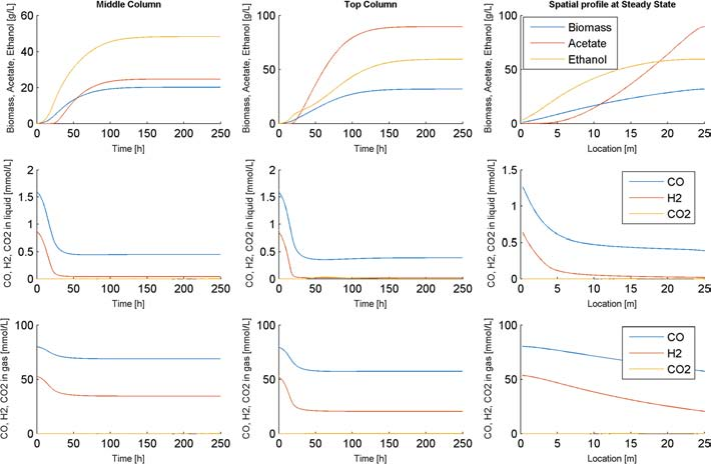
Dynamic simulation of the bubble column reactor model at the nominal operating conditions (Table 1). The first two columns show time resolved predictions at node points in the middle and at the exit of the column, while the third column show spatially resolved predictions for the exit node point at the final time

Our first goal was to investigate the efficiency of DFBAlab for simulating startup of the bubble column reactor with *N* = 100 node points, which yielded a total of 900 ODEs (9 per point) and 600 LPs (6 per point). Despite the substantial computational complexity of this discretized model, we found that a typical 1000 h dynamic simulation for determining a steady-state solution required only about 8 min running DFBAlab and MATLAB version 7.11 on a Dell XPS laptop with Intel Core i7-2760QM processor and 8 GB RAM. Time and spatially resolved predictions obtained for reactor startup with a simulation time of 250 h are shown in Fig. 2. Steady-state conditions were achieved approximately 225 h after startup once the rate of biomass production equaled the rate of biomass removal from the top of the column. The gas and liquid phase CO and H_2_ concentrations decreased along the length of the reactor due to gas consumption, while the biomass, acetate and ethanol concentrations increased along the reactor due to liquid flow. The synthesis of CO_2_ was negligible under these nominal operating conditions. Because the feed gas was relatively rich in CO, the H_2_ conversion was 62 % while the CO conversion was only 29 %. As a result of H_2_ being depleted in the first half of the reactor, considerable acetate was produced in the second half of the reactor and the liquid product stream exiting the top of the column contained more acetate than ethanol (ethanol fraction ~40 %). While we are not aware of any published experimental studies that describe the startup dynamics of syngas bubble columns, our model could be a powerful tool for predicting and optimization reactor startup.

**Fig. 3:**
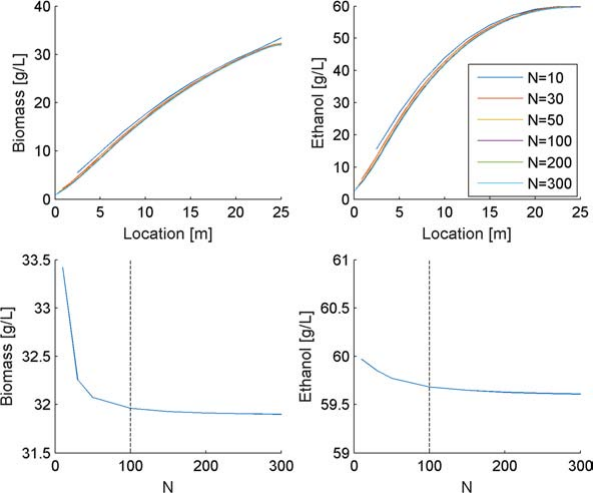
Effect of the number of discretization node points (*N*) on biomass and ethanol concentration spatial profiles (top) and on biomass and ethanol concentrations exiting the reactor (bottom). The chosen value of *N* = 100 is indicated by the dashed lines

To demonstrate that *N* = 100 node points were sufficient to obtain nearly converged solutions, we performed dynamic simulations for reactor startup with different *N* values and compared the resulting steady-state solutions obtained at *t* = 1000 h (Fig. 3). While completely converged solutions appeared to be obtained for 300 node points, this simulation required almost 50 min to complete. For the purposes of this study, we decided that 100 node points provided a suitable compromise between solution accuracy (less than 0.2 % error compared to *N* = 300) and computational time (~8 min per simulation). All remaining simulations were performed with *N* = 100.

**Fig. 4:**
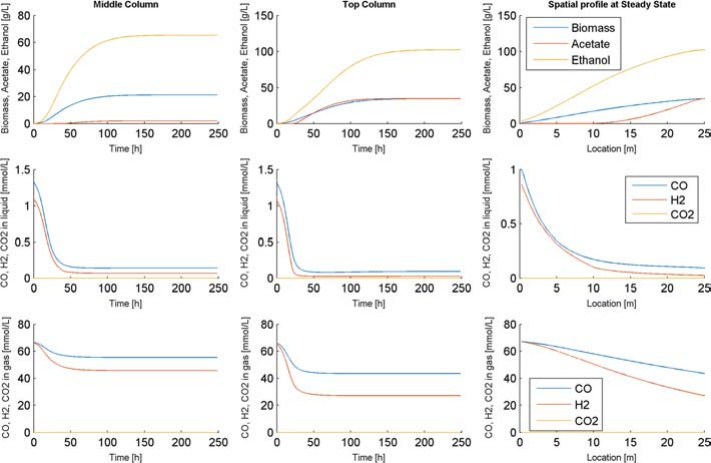
Dynamic simulation of the bubble column reactor model for a CO/H_2_ feed composition of 50/50. The first two columns show time resolved predictions at node points in the middle and at the exit of the column, while the third column show spatially resolved predictions for the exit node point at the final time

To demonstrate the power of our computational framework and to gain insights into bubble column reactor dynamics, we performed additional startup simulations with different parameter values. First we changed the feed composition from the nominal 60/40 CO/H_2_ mixture to a 50/50 CO/H_2_ mixture. The column exhibited similar dynamics for this H_2_ rich feed, as the biomass concentration still required approximately 200 h to reach steady state (Fig. 4). However, the increased H_2_ feed concentration produced a more favorable dissolved H_2_ profile along the column, resulting in an enhanced ethanol titer of 102 g/L and a substantially improved ethanol-acetate ratio of approximately 3 at the reactor outlet once steady state was reached. The amount of biomass produced was not noticeably changed. Due to the increased H_2_ content of the feed, the H_2_ conversion decreased to 60 % and the CO conversion increased to 35 %. Our model predictions were in qualitative agreement with published experimental studies [26–28] showing that hydrogen rich feeds increase both the ethanol titer and the ethanol/acetate split.

**Fig. 5:**
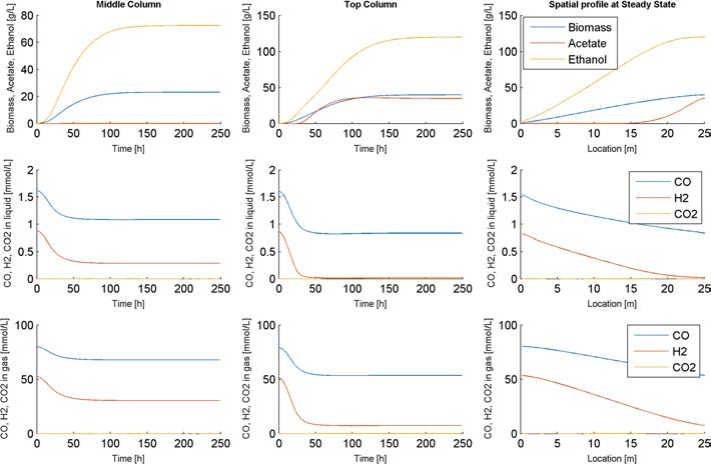
Dynamic simulation of the bubble column reactor model for a CO mass transfer coefficient *k*_*m*,*C*_ = 300 h^−1^. The first two columns show time resolved predictions at node points in the middle and at the exit of the column, while the third column show spatially resolved predictions for the exit node point at the final time. The H_2_ and CO_2_ mass transfer coefficients were set to be 2.5*k*_*m*,*C*_ and *k*_*m*,*C*_, respectively

Next we performed a dynamic simulation with the CO gas–liquid mass transfer coefficient changed from the nominal value *k*_*m,C*_ = 80 h^−1^ to a substantially larger value *k*_*m,C*_ = 300 h^−1^, which could result from syngas microsparging and column internal packing [29]. Consistent with our nominal values (Table 2), we set the H_2_ mass transfer coefficient to be 250 % larger than the CO value and the CO_2_ mass transfer coefficient to equal the CO value. The large increases in gas–liquid mass transfer rates produced faster column dynamics with the biomass concentration requiring only about 150 h to reach steady state (Fig. 5). Once the column reached steady state, the increased mass transfer rates also offered the benefit of increased ethanol titer (120 g/L), a higher ethanol-acetate ratio (3.5) and improved CO (33 %) and H_2_ (86 %) conversions compared to the nominal case. Our predictions were in qualitative agreement with published experimental studies [27, 29, 30] showing that enhanced gas–liquid mass transfer increases gas consumption, the ethanol titer and the ethanol/acetate split.

### Spatiotemporal simulation of a Bacterial Biofilm

Chronic, non-healing wounds are a growing medical challenge associated with diabetes and obesity [31]. These wounds are typically colonized by bacterial species such as *Pseudomonas g*rowing as biofilms on a complex mixture of wound exudate [32, 33]. Bacteria in biofilms can tolerate antimicrobial agent concentrations 10,000 times higher than the same microbes grown planktonically, making treatment of chronic wound biofilms a major challenge [34]. Carbon sources such as glucose are available only from the exudate through the tissue-biofilm interface at the bottom of the biofilm and oxygen is primarily available through the biofilm-air interface at the top of the biofilm. Due to limited diffusion, bacterial biofilms often exhibit strong spatial gradients that affect metabolism, physiology and virulence [35, 36]. The development of predictive metabolic models for simulating these complex spatially structured systems is important to advance understanding and treatment of chronic wound infections.

#### 1. Biofilm model solution

The bacterial biofilm model was formulated by combining a genome-scale metabolic reconstruction of the opportunistic human pathogen *P. aeruginosa* [37] with substrate uptake kinetics and reaction–diffusion equations for substrates and metabolic byproducts. As compared to alternative biofilm modeling approaches based on unstructured intracellular descriptions [38], this model formulation allowed the effects of substrate and byproduct diffusion within the biofilm to be captured with genome-scale resolution. Our preliminary FBA calculations showed that the primary byproducts were acetate and D-alanine. To obtain better agreement with experimental data [39] showing anaerobic succinate secretion by *P. aeruginosa*, we constrained the D-alanine secretion flux to zero such that the only byproducts were acetate and succinate. While other byproducts could be secreted in different biofilm microenvironments, we did not attempt to determine or model other byproducts due to our focus on cellular growth. The spatiotemporal metabolic model was comprised of 5 PDEs for the liquid-phase concentrations of *P. aeruginosa* biomass, glucose, oxygen, acetate and succinate (see Additional file 1: Appendix). Model parameters were obtained from the literature to the extent possible with the remaining parameters specified within reasonable ranges (Table 3). To avoid the complications associated with solving a moving boundary problem, the biofilm was assumed to have a fixed thickness. Therefore, the formulated model was appropriate for predicting the metabolism of *P. aeruginosa* biofilms of a specified thickness rather than predicting the thickness itself. Model simulations show the spatiotemporal dynamics of cellular metabolism within a fixed spatial domain consistent with growth between two stationary surfaces. Steady-state solutions show the spatial distribution of cell and metabolite concentrations within a biofilm of the prescribed thickness. As expected, we found that growth dynamics were strongly affected and steady-state spatial profiles were less affected by the initial cell concentration.

**Table 3:** Parameter values for the *P. aeruginosa* biofilm model

Parameter	Value	Parameter	Value
*L*	100 μm	*v*_*max,O*_	20 mmol/gDW/h
*D*_*GW*_	9.4x10^−6^ cm^2^/s	*K*_*m,O*_	0.003 mmol/L
*D*_*OW*_	26.8x10^−6^ cm^2^/s	*T*	37 °C
*D*_*AW*_	16.2x10^−6^ cm^2^/s	*G*_*b*_	7.5 mmol/L
*D*_*SW*_	12.6x10^−6^ cm^2^/s	*O*_*b*_	0.21 mmol/L
*k*_*G*_ *, k*_*A*_ *, k*_*S*_	2.0x10^−4^ cm/s	*A*_*b*_	0 mmol/L
*k*_*O*_	2.0x10^−2^ cm/s	*S*_*b*_	0 mmol/L
*μ*_*d*_	0.01 h^−1^	*X*_*0*_	1 g/L
*X*_*max*_	200 g/L	*O*_*0*_	0.21 mmol/L
*v*_*max,G*_	10 mmol/gDW/h	*A*_*0*_	0 mmol/L
*K*_*m,G*_	0.5 mmol/L	*S*_*0*_	0 mmol/L

The diffusion terms were discretized using a central difference approximation with second-order accuracy, which produced a set of 5 ODEs in time at each node point. The lexicographic optimization objectives were specified to reflect the known or expected physiology of *P. aeruginosa* (Table 4). We found that the ordering of these objectives had no noticeable effect on predicted biofilm dynamics. Each node point was represented by 5 ODEs for the biomass, glucose, oxygen, acetate and succinate concentrations, 4 algebraic equations for calculating diffusion coefficients as a function of the local biomass concentration [40], and 5 LPs for lexicographic optimization. We used 50 node points for DFBAlab solution with the LP solver Gurobi and the stiff ODE solver ode15s.

**Table 4:** Cellular objective functions used for *P. aeruginosa* metabolism

Number	Direction	Objective	Reason
1	Maximize	Growth rate	Assumed primary objective
2	Minimize	Acetate synthesis rate	Minimize byproduct synthesis
3	Minimize	Succinate synthesis rate	Minimize byproduct synthesis
4	Maximize	Glucose uptake rate	Maximize nutrient consumption
5	Maximize	Oxygen uptake rate	Maximize nutrient consumption

#### 2. Prediction of biofilm physiology

We performed a dynamic simulation for a biofilm thickness of 50 μm with *N* = 50 node points, which produced a discretized model with 250 ODEs (5 per point) and 250 LPs (5 per point). A 1000 h dynamic simulation for determining a steady-state solution required about 15 min on our Dell XPS laptop. This computation time was substantially greater than the 8 min required to simulate the bubble column reactor model over the same time period despite the larger size of the discretized column model (900 ODEs, 600 LPs). While we hypothesize that the increased computation times obtained with the biofilm model were attributable to the diffusion dominated behavior, these results demonstrate the need to better understand the computational complexity of these large-scale ODE/LP systems.

**Figure 6:**
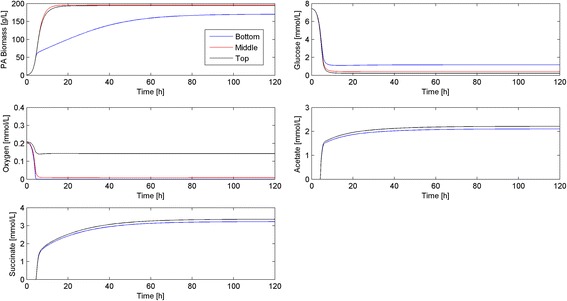
Dynamic simulation of the bacterial biofilm model at the nominal operating conditions (Table 3) and a width *L* = 50 μm. Time resolved predictions are shown for nodes points located at the bottom, middle and top of the biofilm

Figure 6 shows dynamic simulation results for the 50 μm thick biofilm, where time profiles are presented at the bottom (tissue interface), middle and top (air interface) of the biofilm. The bottom layer was characterized by a high glucose concentration, a very low oxygen concentration and a relatively small biomass concentration with slow dynamics. By contrast, the top layer had a very low glucose concentration, a high oxygen concentration and a relatively large biomass concentration with fast dynamics. Experimental studies [9, 41] also have shown the presence of strong spatial gradients in nutrient (e.g. oxygen) levels within biofilms. Despite having a much lower oxygen concentration, the middle layer exhibited similar dynamic and steady-state behavior as the top layer. Spatially uniform acetate and succinate concentrations were obtained throughout the biofilm due to limited removal of the two byproducts at the tissue-biofilm boundary.

**Fig 7:**
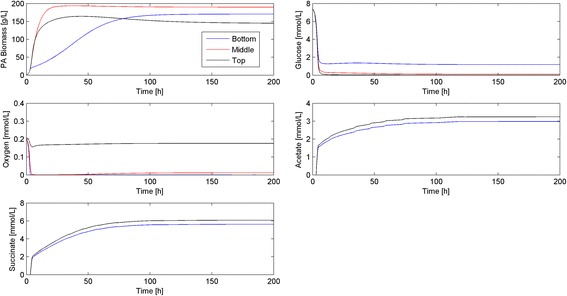
Dynamic simulation of the bacterial biofilm model at the nominal operating conditions (Table 3) and a width *L* = 100 μm. Time resolved predictions are shown for nodes points located at the bottom, middle and top of the biofilm

To explore the impact of biofilm thickness on physiology and to further evaluate our modeling framework, we performed a dynamic simulation for a 100 μm thick biofilm (Fig. 7). This thicker biofilm had slower dynamics, with approximately 200 h required to reach a steady-state solution. Major differences between the 50 and 100 μm thicknesses were observed at the top of the biofilm. In particular, the 100 μm thick biofilm exhibited much slower growth dynamics and less biomass accumulation due to the limited glucose diffusion, a prediction in qualitative agreement with experimental data [36, 42] indicating nutrient limited growth in mature biofilms. The thicker biofilm also produced higher levels of the metabolic byproducts, especially succinate, which could potentially serve as a carbon source for aerobic growth in glucose depleted regimes at the top of the biofilm [43].

**Fig. 8:**
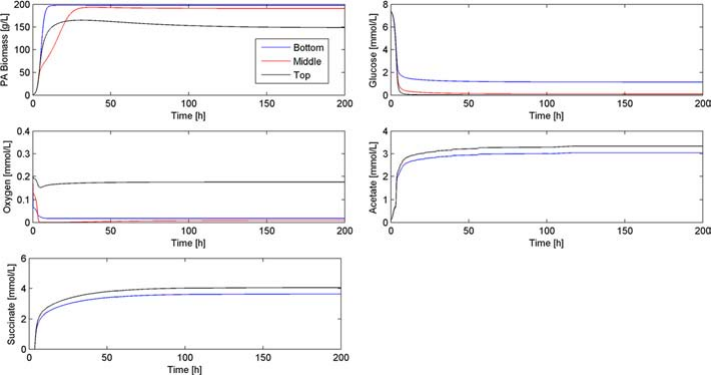
Dynamic simulation of the bacterial biofilm model at the nominal operating conditions (Table 3), width *L* = 50 μm and a mass transfer limited boundary condition with a plasma O_2_ concentration of 0.05 mmol/L imposed at the tissue-biofilm interface. Time resolved predictions are shown for nodes points located at the bottom, middle and top of the biofilm

While the previous simulations were performed assuming the only source of O_2_ was from air at the top of the biofilm, blood plasma has low O_2_ levels [44] that could support limited aerobic growth near the tissue-biofilm interface. To investigate this effect, we modified the boundary condition at *z* = 0 for the O_2_ mass balance (Equation 13 in Additional file 1: Appendix) from a no flux boundary condition to a mass transfer limited boundary condition with a plasma O_2_ concentration of 0.05 mmol/L. The inclusion of an O_2_ source at this interface resulted in a higher O_2_ level, much faster growth dynamics and more biomass accumulation at the bottom of the biofilm (Fig. 8). The establishment of partially aerobic conditions near the tissue-biofilm interface also reduced the overall level of succinate in the biofilm while the acetate level was unaffected.

**Fig. 9:**
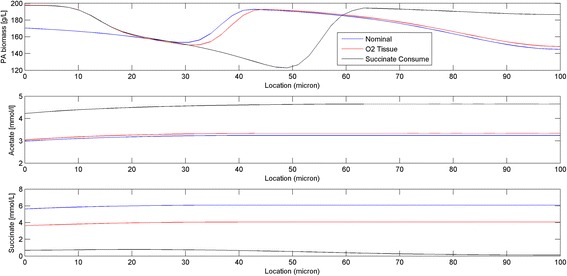
Spatial profiles obtained at the end of 1000 h dynamic simulations for three scenarios: no O_2_ supplied at the tissue-biofilm interface and succinate consumption not allowed (“Nominal”); O_2_ supplied at the tissue-biofilm interface by imposing a mass transfer limited boundary condition with a plasma O_2_ concentration of 0.05 mmol/L but succinate consumption not allowed (“O2 Tissue”); and O_2_ supplied at the tissue-biofilm interface and succinate consumption introduced assuming the same uptake kinetic parameters as used for glucose

Finally we performed a dynamic simulation to investigate the effects of putative succinate reassimilation on biofilm physiology. The thickness was specified as 100 μm and O_2_ was supplied at the tissue-biofilm interface as before. Succinate consumption was included in the model by allowing succinate uptake with the same kinetic parameters as used for glucose (see Equation 11 in Additional file 1: Appendix and Table 3). Figure 9 shows a comparison of steady-state spatial profiles obtained after 1000 h of dynamic simulation for three cases that differ with respect to whether O_2_ was supplied at the tissue-biofilm interface and whether succinate consumption was allowed. If only O_2_ supply at the tissue-biofilm interface was introduced (“O2 Tissue”), the main differences from the nominal case were that more biomass was produced near the interface and lower succinate levels were generated throughout the biofilm. When succinate consumption also was introduced (“Succinate Consume”), then biomass was preferentially accumulated at the top of the biofilm due to succinate oxidation, resulting in a less dense region located in the middle. This prediction was consistent with the known physiology of nutrient limited biofilms [45]. Succinate consumption also resulted in increased acetate levels compared to the other two cases.

## Conclusions

Many natural and engineered microbial systems exist in non-homogeneous environments that require metabolic models that account for both temporal and spatial variations. Our spatiotemporal metabolic modeling framework involves combining genome-scale metabolic reconstructions with fundamental transport equations that govern the relevant convective and/or diffusional processes in the extracellular environment. The PDE model is spatially discretized and the resulting system of ODEs with embedded LPs is integrated using DFBAlab [19], a MATLAB code that performs reliable and efficient dynamic FBA simulations. We demonstrated the capabilities of the method by solving large discretized models for a convection dominated syngas bubble column reactor (900 ODEs, 600 LPs) and a diffusion driven bacterial biofilm model (250 ODEs, 250 LPs). The proposed methodology represents an important step towards rigorously solving spatiotemporal models that combine a genome-scale description of intracellular metabolism and fundamental transport equations for the extracellular environment. A possible limitation of our modeling framework is computational cost, which depends on the number of microbial species, the number of metabolite uptake and secretion fluxes for each species, and the number of node points used for spatial discretization. Consequently, future research will focus on improving computational efficiency including the reduction of genome-scale reconstructions to maintain the same uptake-secretion rate behavior [46] and strategic combination of extracellular byproducts into lumped variables that reduce model size. While our bubble column and biofilm models produce predictions in qualitative agreement with available data, we are currently conducting detailed experimental studies to generate spatially and time resolved data for model validation.

### Availability of supporting data

The datasets supporting the results of this article are included within the article and its additional file.

## Additional file

Additional file 1: Appendix
**The Appendix contains detailed equations for the two spatiotemporal metabolic models studied in this paper: (1) a bubble column reactor for bacterial conversion of synthesis gas to ethanol; and (2) a bacterial biofilm associated with chronic wound infections.** (DOCX 45 kb)

## Abbreviations

DFBAlab: Dynamic flux balance analysis laboratory
FBA: Flux balance analysis
LP: Linear program
ODE: Ordinary differential equation
PDE: Partial differential equation
