# Supplementary material for: Spatiotemporal modeling of microbial metabolism
Source: BMC Syst Biol. 2016 Mar 1;10:21. doi: 10.1186/s12918-016-0259-2 (PMC4774267; doi:10.1186/s12918-016-0259-2)
Supplement: Additional file 1: Appendix — The Appendix contains detailed equations for the two spatiotemporal metabolic models studied in this paper: (1) a bubble column reactor for bacterial conversion of synthesis gas to ethanol; and (2) a bacterial biofilm associated with chronic wound infections. (DOCX 45 kb) [file 12918_2016_259_MOESM1_ESM.docx]

**Appendix**

**Bubble Column Model Equations**

Below we present the balance equations and boundary conditions for the bubble column model. Because our focus was describing spatially varying cellular metabolism rather than detailed modeling of the potentially complex column hydrodynamics [[1](#_ENREF_1)], we assumed ideal plug flow for the vapor phase and plug flow plus axial dispersion for the liquid phase. Convection and dispersion were assumed to occur only in the axial direction of the bubble column reactor such that spatial variations could be captured with a single variable *z*. The gas concentrations at the reactor entrance were calculated from the partial pressures of the feed gas using the ideal gas law. The interested reader is directed to our other paper [[2](#_ENREF_2)] for details concerning the model formulation.

- Dissolved gas uptake kinetics for CO, H_2_ and CO_2_:

$v_{i}=\frac{v_{\max,i}S_{i}}{K_{m,i}+S_{i}}\frac{1}{1+\frac{E_{L}+A_{L}}{K_{I}}}$, (4)

where *S_i_* is the concentration (mmol/L) of the i-th gas phase component, *E_L_* and *A_L_* are the liquid phase concentrations (g/L) of ethanol and acetate, *v_max,i_* is the maximum uptake rate, *K_m,i_* is a saturation constant and *K_I_* is an inhibition constant. The calculated uptakes *v_i_* (mmol/gDW^.^h) are input bounds to the genome-scale metabolic model.

- *C. ljungdahlii* mass balance:

$\begin{matrix} \frac{\partial X(z,t)}{\partial t}=\mu X-\frac{u_{L}}{\varepsilon_{L}}\frac{\partial X}{\partial z}+D_{A}\frac{\partial^{2}X}{\partial z^{2}} \\ \begin{matrix} u_{L}X(0,t)-\varepsilon_{L}D_{A}\frac{\partial X(0,t)}{\partial z}=0 & \frac{\partial X(L,t)}{\partial z}=0 & X(z,0)=X_{0} \end{matrix} \end{matrix}$, (5)

where *t* is time (h), *z* is the length (m) along the column, *X* is the *C. ljungdahlii* biomass concentration (g/L), *D_A_* is the liquid-phase dispersion coefficient and *X_0_* is the initial biomass concentration. The remaining model parameters are defined below Equation 1. The growth rate μ is an output of the genome-scale metabolic model.

- Liquid-phase CO and H_2_ mass balances:

$\begin{matrix} \frac{\partial C_{L}(z,t)}{\partial t}=v_{C}X+\frac{k_{m,C}}{\varepsilon_{L}}(C^{*}-C_{L})-\frac{u_{L}}{\varepsilon_{L}}\frac{\partial C_{L}}{\partial z}+D_{A}\frac{\partial^{2}C_{L}}{\partial z^{2}} \\ \begin{matrix} u_{L}C_{L}(0,t)-\varepsilon_{L}D_{A}\frac{\partial C_{L}(0,t)}{\partial z}=0 & \frac{\partial C_{L}(L,t)}{\partial z}=0 & C_{L}(z,0) \end{matrix}=C_{L0} \\ \frac{\partial H_{L}(z,t)}{\partial t}=v_{H}X+\frac{k_{m,H}}{\varepsilon_{L}}(H^{*}-H_{L})-\frac{u_{L}}{\varepsilon_{L}}\frac{\partial H_{L}}{\partial z}+D_{A}\frac{\partial^{2}H_{L}}{\partial z^{2}} \\ \begin{matrix} u_{L}H_{L}(0,t)-\varepsilon_{L}D_{A}\frac{\partial H_{L}(0,t)}{\partial z}=0 & \frac{\partial H_{L}(L,t)}{\partial z}=0 & H_{L}(z,0)=H_{L0} \end{matrix} \end{matrix}$, (6)

where *C_L_* and *H_L_* are the liquid-phase CO and H_2_ concentrations (mmol/L) and *C_L0_* and *H_L0_* are the initial CO and H_2_ concentrations. The remaining model parameters are defined below Equation 1. The uptake fluxes *v_C_* and *v_H_* are outputs of the genome-scale metabolic model.

- Gas-phase CO and H_2_ mass balances:

$\begin{matrix} \frac{\partial C_{G}(z,t)}{\partial t}=-\frac{k_{m,C}}{\varepsilon_{G}}(C^{*}-C_{L})-\frac{u_{G}}{\varepsilon_{G}}\frac{\partial C_{G}}{\partial z} & C_{G}(0,t)=C_{GF} & C_{G}(z,0)=C_{G0} \\ \frac{\partial H_{G}(z,t)}{\partial t}=-\frac{k_{m,H}}{\varepsilon_{G}}(H^{*}-H_{L})-\frac{u_{G}}{\varepsilon_{G}}\frac{\partial H_{G}}{\partial z} & H_{G}(0,t)=H_{GF} & H_{G}(z,0)=H_{G0} \end{matrix}$, (7)

where *C_G_* and *H_G_* are the gas-phase CO and H_2_ concentrations (mmol/L), respectively, *C_GF_* and *H_GF_* are the gas-phase CO and H_2_ concentrations in the syngas feed stream, and *C_G0_* and *H_G0_* are the initial CO and H_2_ concentrations.

- Liquid-phase ethanol and acetate mass balances:

$\begin{matrix} \frac{\partial E_{L}(z,t)}{\partial t}=M_{E}v_{E}X-\frac{u_{L}}{\varepsilon_{L}}\frac{\partial E_{L}}{\partial z}+D_{A}\frac{\partial^{2}E_{L}}{\partial z^{2}} \\ \begin{matrix} u_{L}E_{L}(0,t)-\varepsilon_{L}D_{A}\frac{\partial E_{L}(0,t)}{\partial z}=0 & \frac{\partial E_{L}(L,t)}{\partial z}=0 & E_{L}(z,0)=E_{L0} \end{matrix} \\ \frac{\partial A_{L}(z,t)}{\partial t}=M_{A}v_{A}X-\frac{u_{L}}{\varepsilon_{L}}\frac{\partial A_{L}}{\partial z}+D_{A}\frac{\partial^{2}A_{L}}{\partial z^{2}} \\ \begin{matrix} u_{L}A_{L}(0,t)-\varepsilon_{L}D_{A}\frac{\partial A_{L}(0,t)}{\partial z}=0 & \frac{\partial A_{L}(L,t)}{\partial z}=0 & A_{L}(z,0)=A_{L0} \end{matrix} \end{matrix}$, (8)

where *E_L_* and *A_L_* are the liquid phase concentrations (g/L) of ethanol and acetate, *M_E_* and *M_A_* are the molecular weights of ethanol and acetate, and *E_L0_* and *A_L0_* are the initial concentrations of ethanol and acetate. The ethanol and acetate secretion fluxes *v_E_* and *v_A_* (mmol/gDw^.^h) are outputs of the genome-scale metabolic model.

- Liquid-phase and gas-phase CO_2_ mass balances:

$\begin{matrix} \frac{\partial D_{L}(z,t)}{\partial t}=v_{D}X+\frac{k_{m,D}}{\varepsilon_{L}}(D^{*}-D_{L})-\frac{u_{L}}{\varepsilon_{L}}\frac{\partial D_{L}}{\partial z}+D_{A}\frac{\partial^{2}D_{L}}{\partial z^{2}} \\ \begin{matrix} u_{L}D_{L}(0,t)-\varepsilon_{L}D_{A}\frac{\partial D_{L}(0,t)}{\partial z}=0 & \frac{\partial D_{L}(L,t)}{\partial z}=0 & D_{L}(z,0)=D_{L0} \end{matrix} \\ \frac{\partial D_{G}(z,t)}{\partial t}=-\frac{k_{m,D}}{\varepsilon_{G}}(D^{*}-D_{L})-\frac{u_{G}}{\varepsilon_{G}}\frac{\partial D_{L}}{\partial z} \\ \begin{matrix} D_{G}(0,t)=D_{GF} & D_{G}(z,0)=D_{G0} \end{matrix} \end{matrix}$, (9)

where *D_L_* and *D_G_* are the liquid and gas phase concentrations (mmol/L) of CO_2_, *D_GF_* is the gas-phase CO_2_ concentration in the syngas feed stream, and *D_L0_* and *D_G0_* are the initial liquid and gas phase CO_2_ concentrations. The CO_2_ secretion flux *v_D_* (mmol/gDw^.^h) is an output of the genome-scale metabolic model.

- Column pressure profile:

$P(z)=P_{L}+\rho_{L}g(L-z)$, (10)

where *P* is the column pressure (Pa), *L* is the column length, *P_L_* is the pressure at the top of the column and ρ_L_ is the density of the liquid-phase assumed to equal the density of water.

**Biofilm Model Equations**

Below we present the balance equations and boundary conditions for the *P. aeruginosa* biofilm model. Only the liquid phase was modeled under the assumptions that oxygen gas-liquid mass transfer was fast compared to oxygen uptake and that acetate and succinate had negligible volatilities. The biofilm width was assumed to be fixed such that complications associated with including a moving boundary could be avoided. Because biofilm expansion was not possible, a maximum biomass concentration was included to avoid unrealistically large biomass concentrations near the glucose rich tissue-biofilm interface. Diffusion was assumed to occur only in the axial direction of the biofilm such that spatial variations could be described with a single variable *z* along the length. Local diffusion coefficients were assumed to depend on the local biomass concentration such that diffusion was reduced in more dense regions of the biofilm [[3](#_ENREF_3)]. Mass transfer resistances at the tissue-biofilm and biofilm-air interfaces were included to restrict glucose and oxygen diffusion, respectively, into the biofilm. Acetate and succinate diffusion out of the biofilm at the tissue-biofilm interface also were restricted.

- Uptake kinetics for glucose and oxygen:

$v_{i}=\frac{v_{\max,i}S_{i}}{K_{m,i}+S_{i}}$, (11)

where *S_i_* is the concentration (mmol/L) of glucose or oxygen, *v_max,i_* is the maximum uptake rate and *K_m,i_* is a saturation constant.

- *P. aeruginosa* mass balance:

$\begin{matrix} \frac{\partial X(z,t)}{\partial t}=\mu X\left(1-\frac{X}{X_{\max}}\right)-\mu_{d}X \\ \begin{matrix} \frac{\partial X(0,t)}{\partial t}=0 & \frac{\partial X(L,t)}{\partial t}=0 & X(z,0)=X_{0} \end{matrix} \end{matrix}$, (12)

where *t* is time (h), *z* is the axial distance (μm) along the biofilm, *L* is the biofilm width, *X* is the *P. aeruginosa* biomass concentration (g/L), μ is the growth rate obtained from the genome-scale metabolic model, μ_d_ is the death rate and *X_0_* is the initial biomass concentration. Because the model lacks a mechanism to restrict cell growth in substrate rich regions of the biofilm due the assumption of a constant thickness, a maximum cell concentration *X_max_* was included to maintain total biomass concentrations within reasonable ranges.

- Glucose and oxygen mass balances:

$\begin{matrix} \frac{\partial G(z,t)}{\partial t}=v_{G}X+D_{G}\frac{\partial^{2}G}{\partial z^{2}} \\ \begin{matrix} -D_{G}\frac{\partial G(0,t)}{\partial z}=k_{G}[G_{b}-G(0,t)] & \frac{\partial G(L,t)}{\partial z}=0 & G(z,0)=G_{0} \end{matrix} \\ \frac{\partial O(z,t)}{\partial t}=v_{O}X+D_{O}\frac{\partial^{2}O}{\partial z^{2}} \\ \begin{matrix} \frac{\partial O(0,t)}{\partial z}=0 & -D_{O}\frac{\partial O(L,t)}{\partial z}=k_{O}[O_{b}-O(L,t)] & O(z,0)=O_{0} \end{matrix} \end{matrix}$, (13)

where *G* and *O* are the glucose and oxygen concentrations (mmol/L), the uptake fluxes *v_G_* and *v_O_* are obtained from the genome-scale metabolic model, *k_G_* is the glucose mass transfer coefficient at the tissue-biofilm interface, *G_b_* is the glucose concentration of the wound exudate, *k_O_* is the oxygen mass transfer coefficient at the biofilm-air interface, *O_b_* is the oxygen concentration of air, and *G_0_* and *O_0_* are the initial glucose and oxygen concentrations. The remaining model parameters are defined below Equation 1.

- Acetate and succinate mass balances:

$\begin{matrix} \frac{\partial A(z,t)}{\partial t}=v_{A}X+D_{A}\frac{\partial^{2}A}{\partial z^{2}} \\ \begin{matrix} -D_{A}\frac{\partial A(0,t)}{\partial z}=k_{A}[A_{b}-A(0,t)] & \frac{\partial A(L,t)}{\partial z}=0 & A(z,0)=A_{0} \end{matrix} \\ \frac{\partial S(z,t)}{\partial t}=v_{S}X+D_{S}\frac{\partial^{2}S}{\partial z^{2}} \\ \begin{matrix} -D_{S}\frac{\partial S(0,t)}{\partial z}=k_{S}[S_{b}-S(0,t)] & \frac{\partial S(L,t)}{\partial z}=0 & S(z,0)=S_{0} \end{matrix} \end{matrix}$, (14)

where *A* and *S* are the acetate and succinate concentrations (mmol/L), the secretion fluxes *v_A_* and *v_S_* are obtained from the genome-scale metabolic model, *k_A_* and *k_S_* are the acetate and succinate mass transfer coefficients at the tissue-biofilm interface, *A_b_* and *S_b_* are the acetate and succinate concentrations of the wound exudate, and *A_0_* and *S_0_* are the initial acetate and succinate concentrations.

- Local biofilm diffusion coefficients:

$\begin{matrix} D_{G}=D_{GW}\left(1-\frac{0.33X^{0.92}}{11.19+0.27X^{0.99}}\right) \\ D_{O}=D_{OW}\left(1-\frac{0.19X^{0.92}}{11.19+0.27X^{0.99}}\right) \\ D_{A}=D_{AW}\left(1-\frac{0.36X^{0.92}}{11.19+0.27X^{0.99}}\right) \\ D_{S}=D_{SW}\left(1-\frac{0.33X^{0.92}}{11.19+0.27X^{0.99}}\right) \end{matrix}$, (15)

where *D_GW_*, *D_OW_*, *D_AW_* and *D_SW_* are the aqueous diffusion coefficients of glucose, oxygen, acetate and succinate at 37 ^o^C.

**References**

1. Kantarci N, Borak F, Ulgen KO. Bubble column reactors. Process Biochem. 2005;40(7):2263-83.

2. Chen J, Gomez JA, Hoffner K, Barton PI, Henson MA. Metabolic modeling of synthesis gas fermentation in bubble column reactors. Biotechnol Biofuels. 2015;8:89.

3. Beyenal H, Tanyolac A, Lewandowski Z. Measurement of local effective diffusivity in heterogeneous biofilms. Water Sci Technol. 1998;38(8-9):171-8.
